# Enhanced Stability of CaO and/or La_2_O_3_ Promoted Pd/Al_2_O_3_ Egg-Shell Catalysts in Partial Oxidation of Methane to Syngas

**DOI:** 10.3390/molecules18078289

**Published:** 2013-07-15

**Authors:** Jinlong Wang, Hongbo Yu, Zhen Ma, Shenghu Zhou

**Affiliations:** 1 Ningbo Institute of Materials Technology & Engineering, Chinese Academy of Sciences, Ningbo 315201, China; 2 Shanghai Key Laboratory of Atmospheric Particle Pollution and Prevention (LAP^3^), Department of Environmental Science and Engineering, Fudan University, Shanghai 200433, China

**Keywords:** palladium catalysts, egg-shell catalyst, partial oxidation of methane, syngas

## Abstract

An egg-shell Pd/Al_2_O_3_ catalyst showed higher activity than a regular Pd/Al_2_O_3_ catalyst in the partial oxidation of methane to syngas, but a common problem of this reaction is the catalyst deactivation on stream. We attempted to modify the egg-shell catalyst via impregnation with some metal oxide additives. Although the addition of MgO did not show any beneficial effect, the addition of CaO and/or La_2_O_3_ significantly improved the stability due to the suppression of carbon deposition and phase transformation of the Al_2_O_3_ support. The catalysts were characterized by X-ray diffraction (XRD), N_2_ adsorption-desorption, and thermogravimetric analysis (TGA).

## 1. Introduction

Synthesis gas, a valuable feedstock for the synthesis of ammonia, methanol, dimethyl ether, acetic acid, *etc*., is mainly produced by steam reforming of methane [1,2,3]. In recent years, increasing attention has been paid to the catalytic partial oxidation of methane (POM) for syngas production because of less energy consumption, lower investment, and more desirable H_2_/CO ratio for some downstream processes [4,5].

The catalysts investigated in the POM processes are mainly supported Ni catalysts and noble metal (e.g., Rh, Pt, Ru, Pd, or Ir) catalysts [6,7]. Ni-based catalysts are cheap, but they deactivate easily due to carbon deposition and sintering above 700 °C [8]. Noble metal-based catalysts have much higher activity, and are less susceptible to catalyst deactivation [9,10].

Among noble metal catalysts, Pd catalysts supported on Al_2_O_3_, SiO_2_, MgO, CaO, and rare earth metal oxides have been used in POM [11,12]. The catalytic activity and stability can be improved by adding some promoters. For instance, Ryu *et al*. investigated the addition of CeO_2_, BaO, and SrO on the catalytic performance of Pd/Al_2_O_3_ for POM, and found that the optimum catalyst was a triply promoted Pd catalyst, namely Pd(2)/CeO_2_(23)/BaO(11)/SrO(0.8)/Al_2_O_3_ [13].

Because POM is performed under very high space velocity, the inner diffusion and reaction inside the pores of the catalysts are limited [14]. Therefore, it is desirable to disperse the active species on the outer surface of the catalyst as far as possible. Egg-shell catalysts can satisfy such a need. Besides, the cost of catalysts can be significantly lowered when adopting the egg shell design. In recent years, Rh [15,16] and Ni [14,17,18] egg-shell catalysts have been investigated in POM. For instance, Chen and co-workers compared the performance of egg-shell and uniform Ni/Al_2_O_3_ catalysts in POM, and found that egg-shell Ni/Al_2_O_3_ was more active due to the distribution of nickel component in the outer region of particles [17].

Herein, a Pd/Al_2_O_3_ egg-shell catalyst and its modified versions were tested in POM. The Pd/Al_2_O_3_ egg-shell catalyst showed higher activity than a conventional Pd/Al_2_O_3_, and the addition of CaO and/or La_2_O_3_ additives can significantly improve the stability on stream due to suppressed carbon deposition and phase transformation of the Al_2_O_3_ support.

## 2. Results and Discussion

**Figure 1:**
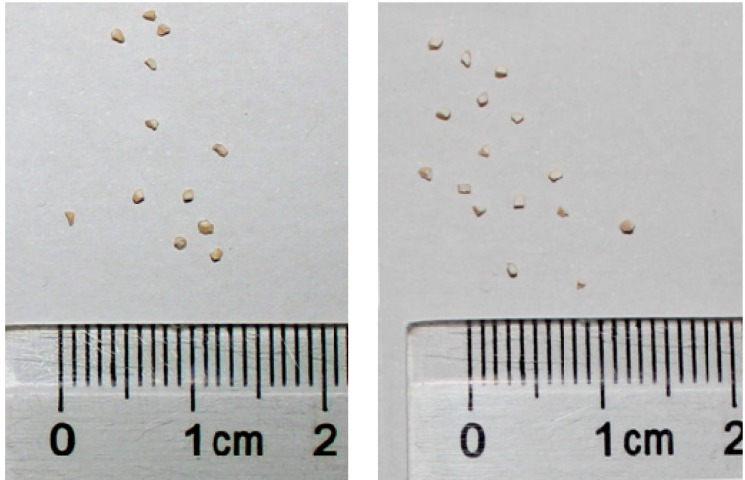
Photos showing the cross section of the prepared egg-shell catalysts. Left: PdO/Al_2_O_3_; Right: PdO/La_2_O_3_/CaO/Al_2_O_3_.

Figure 1 shows the photos of the prepared egg-shell catalysts before reduction, highlighting the cross section. 

The outer surface was dark brown and the inner part of the particles was white, suggesting the accumulation of Pd species on the catalyst surface. The average thickness of the Pd-rich eggshell was approximately 0.2 mm. The catalysts after reduction also showed the egg-shell structure, as seen from the grey-pale black color of the shell while white color at the center.

Table 1 summarizes the specific surface areas of support (calcined at 1,000 °C) and egg-shell catalyst samples reduced at 750 °C. The surface area of Pd/Al_2_O_3_ egg-shell catalyst was lower than its corresponding Al_2_O_3_ support, probably due to the blockage of the pores by loading Pd as well as some degradation of the support as a result of the catalyst preparation procedure [19]. The addition of MgO, CaO, La_2_O_3_, or CaO and La_2_O_3_ into Pd/Al_2_O_3_ could retain a higher surface area.

**Table 1:** Specific surface areas of support (calcined at 1,000 °C) and egg-shell catalyst samples reduced at 750 °C.

Sample	Specific surface area (m^2^/g)
Al_2_O_3_	118
Pd/Al_2_O_3_	58
Pd/MgO/Al_2_O_3_	99
Pd/CaO/Al_2_O_3_	78
Pd/La_2_O_3_/Al_2_O_3_	78
Pd/La_2_O_3_/CaO/Al_2_O_3_	102

**Figure 2:**
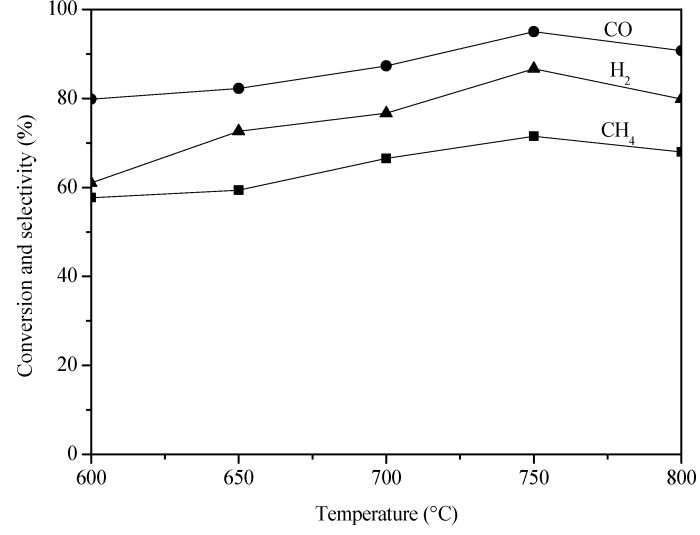
Catalytic performance of Pd/Al_2_O_3_ egg-shell catalyst in POM reaction at different temperatures.

Figure 2 shows the performance of Pd/Al_2_O_3_ egg-shell catalyst in POM reaction as a function of reaction temperature. 

The CH_4_ conversion increased with the reaction temperature and reached 72% at 750 °C. For comparison, the CH_4_ conversion on a regular Pd/Al_2_O_3_ catalyst with the same Pd loading (0.2%) was 49% at 750 °C, demonstrating the advantage of the egg shell design. The CH_4_ conversion on Pd/Al_2_O_3_ egg-shell catalyst decreased when the reaction temperature was above 750 °C, due to the accelerated carbon deposition at high temperatures.

**Figure 3:**
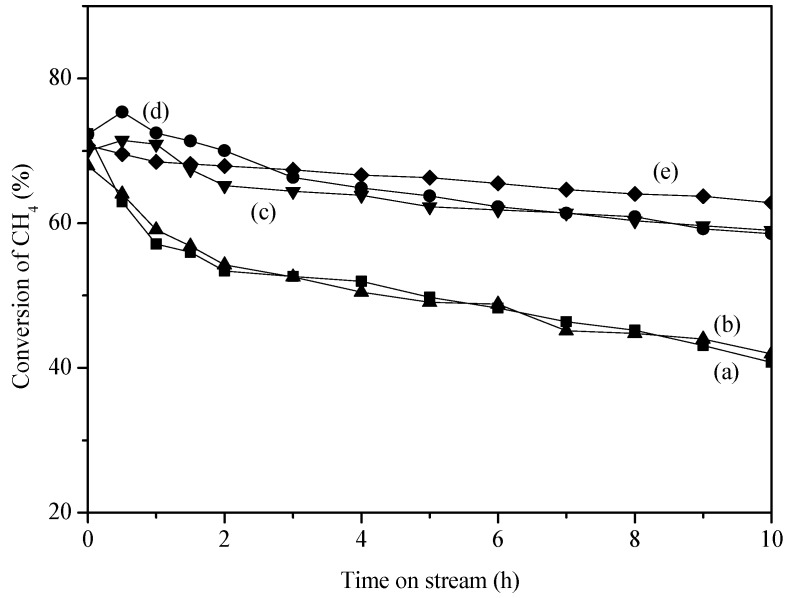
Performance of the egg-shell catalysts as a function of time on stream in POM reaction. (**a**) Pd/Al_2_O_3_; (**b**) Pd/MgO/Al_2_O_3_; (**c**) Pd/CaO/Al_2_O_3_; (**d**) Pd/La_2_O_3_/Al_2_O_3_; (**e**) Pd/La_2_O_3_/CaO/Al_2_O_3_.

Figure 3 shows the CH_4_ conversion on various egg-shell catalysts at 750 °C as a function of time on stream. The initial CH_4_ conversions on these catalysts were comparable, ranging from 67% to 73%. However, the deactivation behaviors were quite different. Pd/Al_2_O_3_ egg-shell catalyst deactivated quickly on stream, as seen by the dropping of CH_4_ conversion from 72% to 40% after operation for 10 h. The addition of MgO barely changed the quick deactivation, whereas the addition of CaO and/or La_2_O_3_ significantly suppressed the deactivation. In particular, Pd/La_2_O_3_/CaO/Al_2_O_3_ egg-shell catalyst showed the best stability: the CH_4_ conversion changed from 70% to 62% after operation for 10 h. 

**Figure 4:**
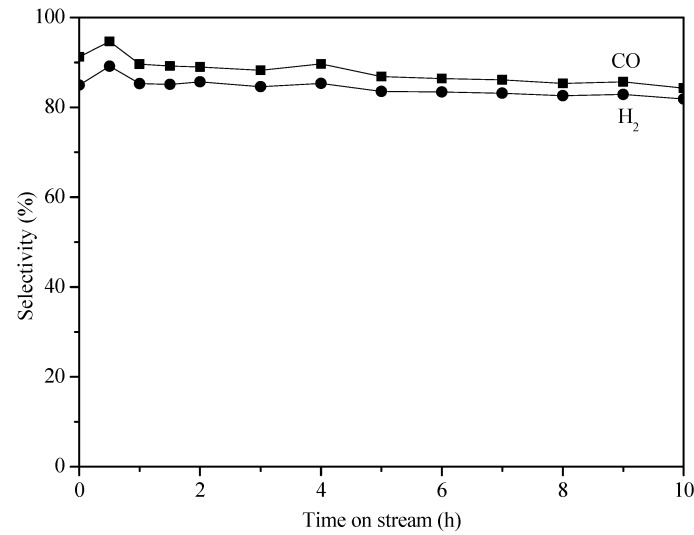
Selectivity to CO and H_2_ on Pd/La_2_O_3_/CaO/Al_2_O_3_ egg-shell catalyst in POM reaction.

Figure 4 shows the H_2_ selectivity and CO selectivity on Pd/La_2_O_3_/CaO/Al_2_O_3_ egg-shell catalyst as a function of time on stream. The H_2_ selectivity and CO selectivity were about 85% and 91%, respectively.

**Figure 5:**
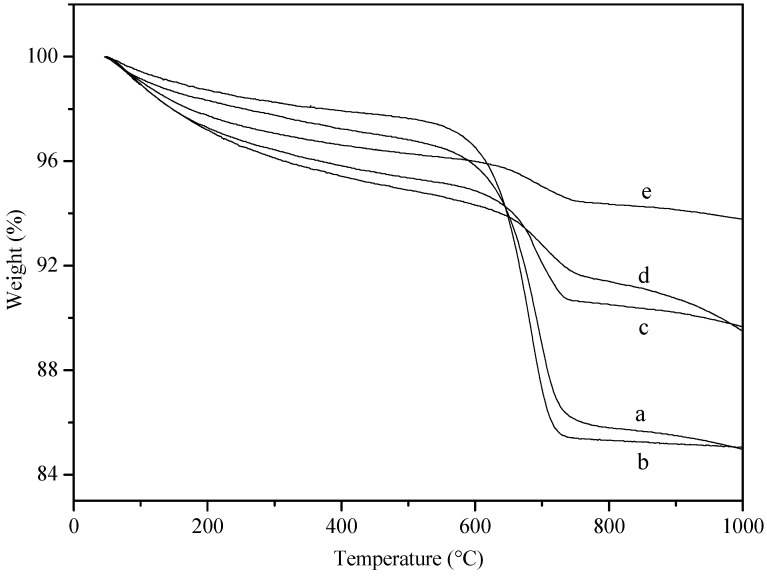
TG curves of the spent egg-shell catalysts collected after operation for 10 h. (**a**) Pd/Al_2_O_3_; (**b**) Pd/MgO/Al_2_O_3_; (**c**) Pd/CaO/Al_2_O_3_; (**d**) Pd/La_2_O_3_/Al_2_O_3_; (**e**) Pd/La_2_O_3_/CaO/Al_2_O_3_.

One reason for catalyst deactivation in the POM process is the deposition of carbon [20]. Indeed, the outer surface of the spent catalysts turned into black, indicating the deposition of carbon during the course of reaction. Therefore, TGA was employed to characterize the spent catalysts. As shown in Figure 5, all the samples showed an obvious weight loss from ca. 600 °C to 750 °C, attributing to the oxidation of deposited carbon, most likely graphitic carbon [21]. Interestingly, the amounts of deposited coke were different with different spent egg-shell catalysts: those on Pd/CaO/Al_2_O_3_ (4.3%), Pd/La_2_O_3_/Al_2_O_3_ (3.4%), and Pd/La_2_O_3_/CaO/Al_2_O_3_ (2.0%) were much less than those on Pd/Al_2_O_3_ (10.7%) and Pd/MgO/Al_2_O_3_ (12.1%), correlating nicely with the deactivation behaviors.

**Figure 6:**
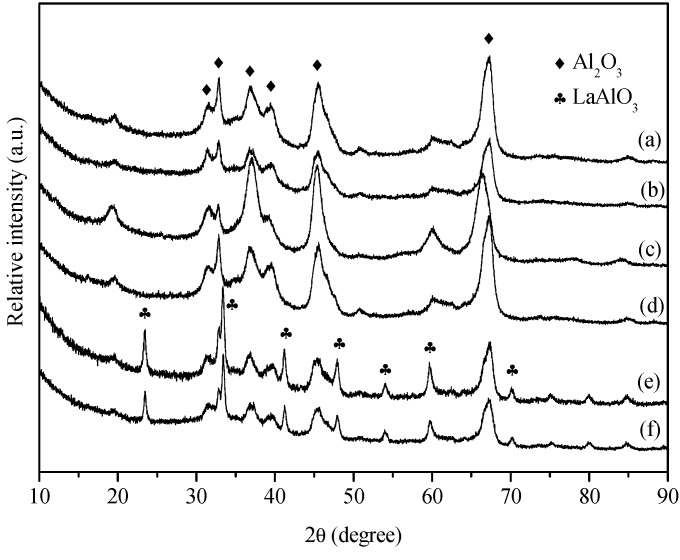
XRD patterns of support and egg-shell catalysts reduced at 750 °C. (**a**) Al_2_O_3_; (**b**) Pd/Al_2_O_3_; (**c**) Pd/MgO/Al_2_O_3_; (**d**) Pd/CaO/Al_2_O_3_; (**e**) Pd/La_2_O_3_/Al_2_O_3_; (**f**) Pd/La_2_O_3_/CaO/Al_2_O_3_.

Another reason for catalyst deactivation in POM process is the phase transformation of Al_2_O_3_ [22]. Figure 6 shows the XRD patterns of the support (calcined at 1,000 °C) and egg-shell catalysts reduced at 750 °C. The characteristic diffraction peaks of Al_2_O_3_ support calcined at 1,000 °C (Figure 6a) were at 31.5°, 32.9°, 36.8°, 39.8°, 45.6°, and 67.3°, corresponding to δ phase (JCPDS 4-877). For Pd/Al_2_O_3_ catalyst, Pd diffractions were not found since its loading was low (0.2%) and only Al_2_O_3_ peaks were visible (Figure 6b). Pd/MgO/Al_2_O_3_ (Figure 6c) showed characteristic diffraction peaks shifted to the direction in lower values of 2θ due to the substitution of Al^3+^ by Mg^2+^ [23]. Pd/CaO/Al_2_O_3_ (Figure 6d) showed no diffraction peaks of CaO, indicating the high dispersion of CaO in the corresponding catalyst. Peaks at 23.5°, 33.4°, 41.2°, 47.9°, 54.1°, 60.0°, 70.1° in the pattern of Pd/La_2_O_3_/Al_2_O_3_ (Figure 6e) were ascribed to the formation of LaAlO_3_ (JCPDS 31-22) at high temperatures [24]. The characteristic diffraction peaks of Pd/La_2_O_3_/CaO/Al_2_O_3_ (Figure 6f) were similar to those of Pd/La_2_O_3_/Al_2_O_3_ (Figure 6e), again without the peaks of CaO species.

**Figure 7:**
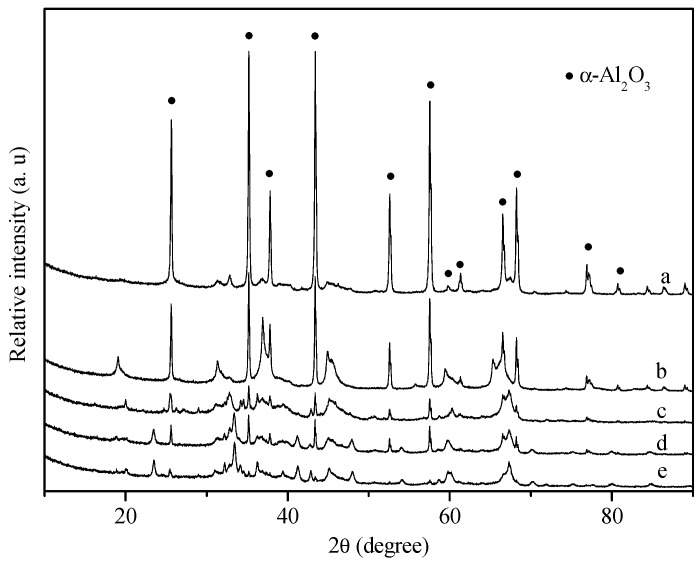
XRD patterns of spent egg-shell catalysts collected after operation for 10 h. (**a**) Pd/Al_2_O_3_; (**b**) Pd/MgO/Al_2_O_3_; (**c**) Pd/CaO/Al_2_O_3_; (**d**) Pd/La_2_O_3_/Al_2_O_3_; (**e**) Pd/La_2_O_3_/CaO/Al_2_O_3_.

Figure 7 shows the XRD patterns of spent egg-shell catalysts. The diffraction peaks due to α-Al_2_O_3_ (JCPDS 10-0173) of the used Pd/Al_2_O_3_ catalyst appeared as shown in Figure 7a. Meanwhile, the diffraction peaks attributed to δ-Al_2_O_3_ became weaker, indicating the partial transformation from δ-Al_2_O_3_ into α-Al_2_O_3_ phase during POM. The addition of Mg slightly impeded the phase transformation of Al_2_O_3_, whereas the addition of CaO and/or La_2_O_3_ dramatically inhibited the phase transformation of Al_2_O_3_. The inhibition of phase transformation of Al_2_O_3_ by addition of La was probably due to the formation of LaAlO_3_ in Al_2_O_3_ surface. Chen and co-workers found that the deactivation of egg-shell Ni/MgO-Al_2_O_3_ catalyst in POM was due to the formation of NiO-MgO solution and/or NiAl_2_O_4_, phase transformation, and sintering [14].

## 3. Experimental

### 3.1. Catalyst Preparation

Preparation of PdO/Al_2_O_3_, PdO/MgO/Al_2_O_3_, and PdO/CaO/Al_2_O_3_ egg-shell samples: γ-Al_2_O_3_ Raschig rings purchased from Yixing YiPu Catalyst Company (Yixing, China) were pre-calcined at 1,000 °C for 2 h and then crushed to 1.0–1.3 mm particles. Al_2_O_3_ particles (5 g) were impregnated with an aqueous solution of Pd precursor by the previously described preparation method [19]. The obtained materials were calcined at 500 °C for 2 h to obtain PdO/Al_2_O_3_. The PdO/Al_2_O_3_ samples were impregnated with an aqueous solution of Mg(NO_3_)_2_ or Ca(NO_3_)_2_ at room temperature by an incipient impregnation method, and then calcined at 500 °C for 2 h to obtain PdO/MgO/Al_2_O_3_ and PdO/CaO/Al_2_O_3_, respectively. For the preparation of PdO/La_2_O_3_/Al_2_O_3_ and PdO/La_2_O_3_/CaO/Al_2_O_3_, pre-calcined Al_2_O_3_ particles (5 g) were impregnated with an aqueous solution of La(NO_3_)_3_ and then calcined to obtain La_2_O_3_/Al_2_O_3_ supports. The supports were impregnated with an aqueous solution of Pd precursor by the preparation method provided by [19] and then calcined at 500 °C for 2 h for the preparation of egg-shell PdO/La_2_O_3_/Al_2_O_3_ samples. The above PdO/La_2_O_3_/Al_2_O_3_ was impregnated with an aqueous solution of Ca(NO_3_)_2_, and then calcined at 500 °C for 2 h to obtain PdO/La_2_O_3_/CaO/Al_2_O_3_. The Pd loading contents were 0.2% (wt %) in all catalysts. The weight ratios of the additive La, Mg, and Ca to Al_2_O_3_ were 0.1, 0.05, and 0.05, respectively.

### 3.2. Catalyst Characterization

The powder X-ray diffraction (XRD) patterns were recorded on a Bruker Advance diffractometer (Bruker, Karlsruhe, Germany) with a Cu Kα monochromatic radiation operated at 40 kV and 40 mA, using a scanning range 2θ between 10° and 90°.

The BET surface areas of the support materials and catalysts were measured by N_2_ adsorption at −196 °C using a Micromeritics ASAP 2020 instrument (Micromeritics, Norcross, GA, USA). The samples were outgassed under N_2_ flow at 300 °C for 2 h before the measurements.

The amount of carbon deposited on the catalysts after reaction was determined on a Pyris Diamond TG instrument (PerkinElmer, Waltham, MA, USA) by heating the samples from room temperature to 1,000 °C (heating rate 10 °C/min) in a flowing air (25 mL/min).

### 3.3. Catalytic Test

The POM reaction was carried out in a continuous flow reactor apparatus equipped with a quartz tube reactor operated at atmospheric pressure. The flow rate of gases was controlled by mass flow controllers. The catalysts (0.5 g) were firstly reduced in a 60 mL/min H_2_ flow at 750 °C for 30 min, then a 60 mL/min Ar flow was introduced to flush the system. Afterwards, a mixture of CH_4_ (99.9%, 277.8 mL/min) and O_2_ (99.999%, 138.9 mL/min) with a molar ratio of 2:1 at weight hour space velocity (WHSV) of 5 × 10^4^ mL·g^−^^1^·h^−^^1^ was introduced. The product gases were analyzed online by a gas chromatograph equipped with a TCD detector and TDX-01 packed column. Water was trapped before gases entered the gas chromatograph.

## 4. Conclusions

A series of Pd/Al_2_O_3_-based egg-shell catalysts were tested in the partial oxidation of methane. The addition of MgO, CaO, or La_2_O_3_ additives had no dramatic effect on the initial CH_4_ conversion. The addition of MgO additive had no effect on the catalyst stability, whereas the addition of CaO and/or La_2_O_3_ significantly improved the catalyst stability because they suppressed the formation of carbon deposit and phase transformation of the Al_2_O_3_ support. More experiments should be done in the future to understand why the addition of certain additives could suppress the deposition of carbon.
